# A novel mutation in nuclear prelamin a recognition factor-like causes diffuse pulmonary arteriovenous malformations

**DOI:** 10.18632/oncotarget.13156

**Published:** 2016-11-07

**Authors:** Hong-zhou Liu, Chun-xian Du, Jing Luo, Xue-ping Qiu, Zu-hua Li, Qi-yong Lou, Zhan Yin, Fang Zheng

**Affiliations:** ^1^ Center for Gene Diagnosis, Zhongnan Hospital of Wuhan University, Wuhan, Hubei, 430071, China; ^2^ Department of Pneumology, Zhongnan Hospital of Wuhan University, Wuhan, Hubei, 430071, China; ^3^ Key Laboratory of Aquatic Biodiversity and Conservation of Chinese Academy of Sciences, Institute of Hydrobiology, Chinese Academy of Sciences, Wuhan 430072, Hubei, China

**Keywords:** pulmonary arteriovenous malformations, capillary malformations, nuclear prelamin A recognition factor-like, whole exome sequencing, zebrafish

## Abstract

Two daughters in a Chinese consanguineous family were diagnosed as diffuse pulmonary arteriovenous malformations (PAVMs) and screened using whole exome sequencing (WES) and copy number variations (CNVs) chips. Though no mutation was found in the established causative genes of capillary malformation-AVMs (CM-AVMs) or PAVMs, Ser161Ile (hg19 NM_022493 c.482G>T) mutation in nuclear prelamin A recognition factor-like (NARFL) was identified. Ser161Ile mutation in NARFL conservation region was predicted to be deleterious and absent in 500 population controls and Exome Aggregation Consortium (ExAC) Database. And there was a dosage effect of the mutation on mRNA levels among family members and population controls, consistent with the instability of mutant mRNA in vitro. Accordingly, in lung tissue of the proband, NARFL protein expression was reduced but Fe^3+^ was overloaded with vascular endothelial growth factor (VEGF) overexpression. Furthermore, NARFL-knockdown cell lines demonstrated decreased activity of cytosolic aconitase, while NARFL-knockout zebrafish presented ectopic subintestinal vessels sprouts and upregulated VEGF. So we concluded that the Ser161Ile mutant induced NARFL deficiency and eventually diffuse PAVMs probably through VEGF pathway. In a word, we detected a functional mutation in NARFL, which might be the pathogenic gene in this pedigree.

## INTRODUCTION

Pulmonary arteriovenous malformations (PAVMs) have the latest incidence of ~1 in 2600 based on the diagnosis with the thoracic computed tomography (CT) [1]. PAVMs are abnormal vessels diseases, as fistulous connections between pulmonary arteries and veins [2]. Diffuse PAVMs are recorded when PAVMs are involved in every subsegmental artery within at least one lobe [2-3]. Capillary malformations (CM), another kind of vessels disease, can be an isolated clinical disease or associated with syndromic vascular anomalies [4], such as Sturge-Weber, Klippel-Trenaunay, Parkes-Weber, CM-AVMs [5-6], macrocephaly-CM (M-CM), and diffuse CM with overgrowth (DCMO) [3]. Among them, CM-AVMs as capillary level PAVMs, are a newly recognized clinical entity [5-7]. So far, only *Endoglin* (*ENG*), *activin A receptor type II-like 1* (*ACVRL1*), *Mothers against decapentapledic homolog 4* (*SMAD4*), and *RAS activator 1* (*RASA1)* were reported to be causal genes of PAVMs and CM-AVMs [7-11].

Here, we describe a family where two female offsprings of first cousin parents presenting diffuse PAVMs. To identify the causative mutations, whole exome sequencing (WES) and copy number variations (CNVs) chips were performed in the two daughters and the parents. And functional studies were carried on the identified possible causal gene *nuclear prelamin A recognition factor-like (NARFL)*.

NARFL was also known as iron-only hydrogenase-like protein 1 (IOP1) [12]. It was a ubiquitous iron-sulfur (Fe-S) protein in every living cell and played an essential role in Fe-S protein biogenesis in many physiologic processes, including electron transport, enzyme catalysis, and Krebs cycle [13-14].

## RESULTS

### Clinical diagnosis

**Figure 1:**
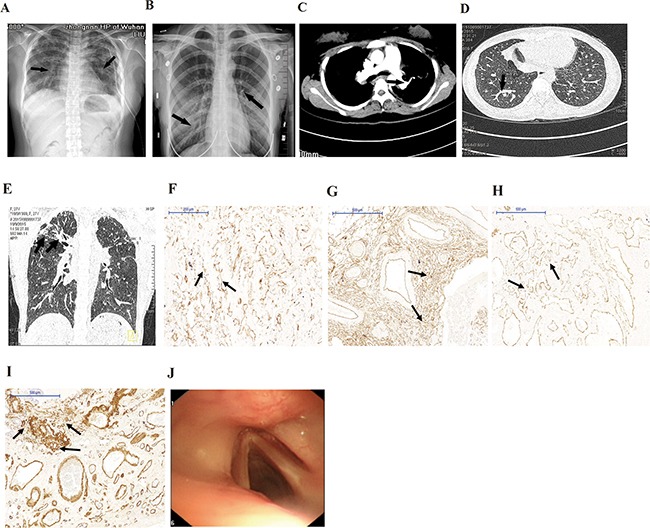
Clinical characteristics of patients **A, B.** X-ray showed ground glass appearance (arrows) in lungs of the proband (A) and the sister (B). **C.** Enhanced CT scans showed AVMs in lungs of the proband. **D, E.** CT scans showed AVMs and diffuse pulmonary interstitial inflammation (arrows) in the proband (D) and the sister (E). **F.** Immunostaining for CD31 showed diffuse vascular malformation (arrows) without any recognizable architectural structure in lung biopsies of the proband (×100). **G, H**. and **I.** Immunostaining for CD34 (G), SMA (H), and CK (I) showed diffuse vascular malformation (arrows) without any recognizable architectural structure in lung biopsies of the proband (×50). **J.** Fiberoptic bronchoscopy showed no telangiectases or other dermal lesions.

The 20-year-old proband was admitted to Center for Gene Diagnosis, Zhongnan Hospital of Wuhan University with cyanosis, dyspnea, clubbing, voice hoarse, and severe hyoxemia. Echocardiography revealed pulmonary arterial hypertension (PAH). Arterial blood gas analysis revealed right-to-left shunting (pH=7.457, PaO_2_=348mmHg (<500mmHg)). Chest X-ray and computed tomography (CT) scan showed a ground glass appearance indicating inflammation (Figure 1A). Enhanced CT scan showed pulmonary arteriovenous malformation (Figure 1C). Lung biopsies showed diffuse PAVMs (Figure 1F, 1G, 1H, 1I). The proband first appeared dyspnea on exertion at 12-year-old. Then she had hemoptysis and was diagnosed as TB infection followed by anti-TB drugs treatment for seven months at 18-year-old. After the proband suffered from progressive dyspnea for one year, she was diagnosed as diffuse PAVMs at 20-year-old mainly based on lung biopsies. Eventually, she died of pulmonary hypertension and cardiac and respiratory failure despite treatment with 50mg steroids for one month at 22-year-old. The proband's elder sister also had occasional hemoptysis, dyspnea on exertion, clubbing and voice hoarse. She was diagnosed as diffuse PAVMs mainly based on imageology results (Figure 1B, 1D, 1E).

### Identification of novel mutations

**Figure 2:**
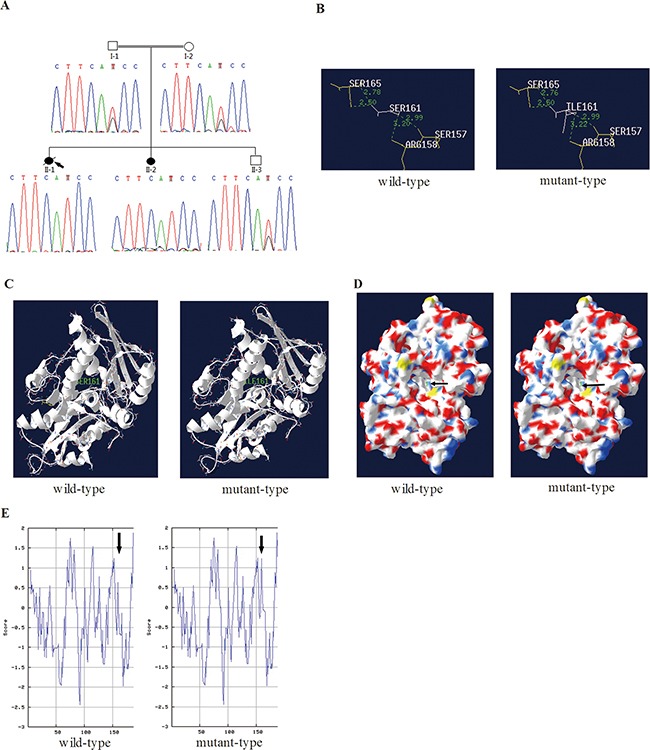
In silico prediction of mutant NARFL **A.** Sequence chromatograms showed the mutation Ser161Ile in NARFL of the family members. **B.** The distance of hydrogen bonds in the NARFL (Ser161Ile) was increased (from 3.20 to 3.22). **C.** The helix was unaffected in the secondary structure of the Ser161Ile. **D.** The Ser161 was in cavity (arrows) of NARFL in the tertiary structure, which might be active center. **E.** Hydropathicity and hydrophobicity of the Ser161Ile were affected.

The entire list of candidate variants was listed in Supplementary Table S1 online and only 1737 protein-altering variants were considered [15]. Then the variants were filtered against BGI-Shenzhen four inner databases, dbSNP137, HapMap8 and 1000 human genome databases and only 459 variants were considered. Filter variants were predicted by the SIFT software and 110 variants were considered because of damaging prediction. Among the 110 variants, 4 variants of genes which were ubiquitously expressed, such as in vessels and lungs according to the Uniprot. Only Ser161Ile (hg19 NM_022493 c.482G>T) in *NARFL* gene co-segregated with the phenotype in the family and was not present in Exome Aggregation Consortium (ExAC) Database (http://exac.broadinstitute.org). The mutation is a polar amino acid (Ser) replaced by the nonpolar (Ile) in the 161st residue of NARFL, which is highly conserved in evolution. The mutation was confirmed by Sanger sequencing (Figure 2A) and absent in 500 population controls. No candidate CNVs according to the autosomal recessive model were found and were reported to be associated with PAVMs and CM-AVMs (Supplementary Table S2 online). Furthermore, *NARFL* gene of thirty five sporadic patients with similar symptoms was sequenced and one new polymorphism of Leu299Met (hg19 NM_022493 c.895C>A) was found.

### In silico prediction of mutant NARFL proteins

The Ser161Ile (hg19 NM_022493 c.482G>T) in NARFL was predicted to be deleterious using SIFT and Polyphen-2. Furthermore, in the Ser161Ile mutant, the distance of hydrogen bond between Ser161 (Ile161) and Arg158 increased from 3.20 to 3.22 (Figure 2B) in the cavity of NARFL protein (Figure 2D). However, no change of the helix at position 161 was observed (Figure 2C). And the hydropathicity and hydrophobicity of mutant NARFL (Ser161Ile) were affected slightly (Figure 2E). Considering that in silico prediction of the structure change of mutant proteins was slight, we hypothesized this novel mutation Ser161Ile might affect the *NARFL* mRNA level.

### Decreased level and stability of the mutant NARFL mRNA

**Figure 3:**
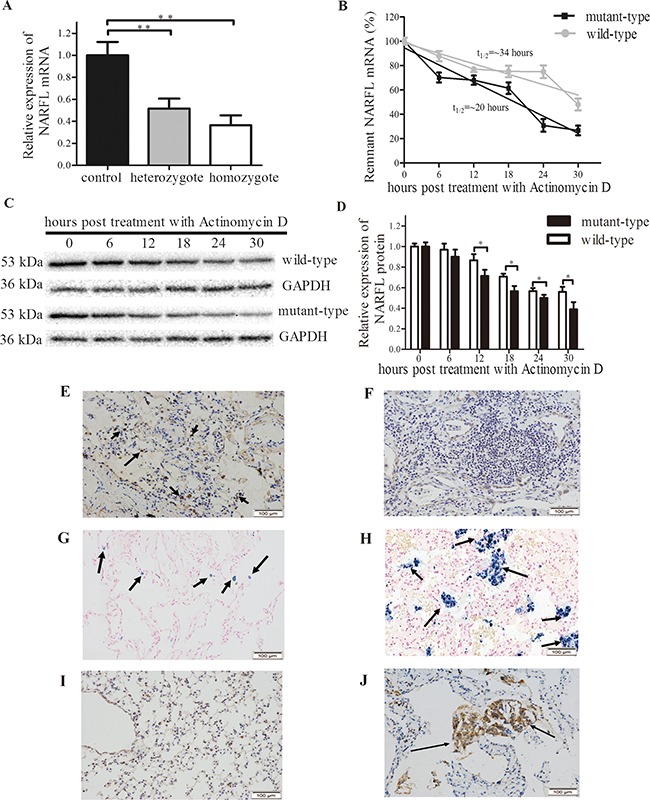
NARFL Expression in family members and results of mRNA stability assay **A.** *NARFL* mRNA levels in heterozygote (n=3) and homozygote (n=2) were significantly lower than population controls (n=6). **B.** Mutant *NARFL* mRNA level decreased more sharply than wild type *NARFL* after actinomycin D treatment. The *NARFL*/*GAPDH* ratio was expressed as a percent of the value obtained for 0 hour post-treatment and then plotted as the semi-log to reflect the difference of mRNA decay rate. **C.** Mutant NARFL protein levels decreased more sharply than wild-type NARFL after actinomycin D treatment. **D.** NARFL protein levels were recorded as gray scale values and analyzed using Image J software. **E, F**. NARFL was examined using immunohistochemical staining, and strong expression of NARFL (indicated by arrows) was observed in the lung tissues of the controls (E) compared to the proband (F) (n = 6, *p* = 0.002). **G, H**. Lower level of Fe^3+^ (indicated by arrows) was observed in controls (G) compared to the proband (H) (n=6, *p* = 0.0009). **I, J**. Lower expression level of VEGF (indicated by arrows) was observed in controls (I) compared to the proband (J) (n=6, *p* = 0.0001). Images E-J are ×200 magnification. (*, *p* < 0.05; **, *p* < 0.01)

There was a decrease in mutant *NARFL* mRNA levels in vivo (Figure 3A). The sisters were homozygotes and their relative expression levels of *NARFL* mRNA decreased by 0.36-fold, while the parents and the brother were heterozygotes with relative expression levels of 0.51-fold, compared with population controls (*p* < 0.01).

Sequentially, as shown in vitro, mRNA levels of Ser161Ile mutant (t_half-time_ = 20 hours) dropped significantly in transfected cells, compared with the wild-type (t_half-time_ = 34 hours) (*p* < 0.05, *n*=3, Figure 3B). And this phenomenon was also observed in protein levels (Figure 3C, 3D). These indicated that *NARFL* mRNA levels were declined in mutation carriers probably because of mRNA instability. Accordingly, the NARFL protein expression in the proband's lung tissue was significantly reduced compared with controls (*p* = 0.002, *n* = 6, Figure 3E, 3F). In addition, the Fe^3+^ overload and vascular endothelial growth factor (VEGF) overexpression were both observed in the proband's lung tissue compared with controls (*p* = 0.0009, *n* = 6 & *p* = 0.0001, *n* = 6, Figure 3G, 3H, 3I, 3J).

### NARFL knockdown induced declined cytosolic Fe-S protein aconitase activity in cells

**Figure 4:**
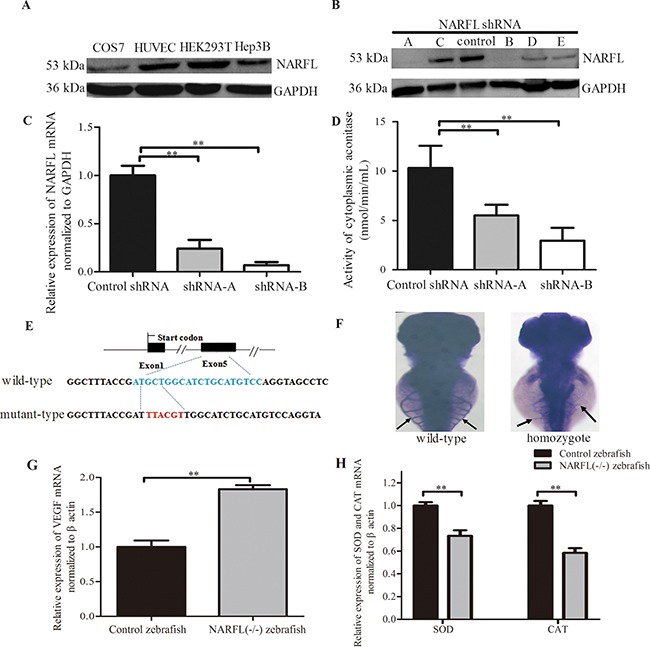
Effects of NARFL-knockdown in HEK293T cells and NARFL-knockout in zebrafish **A.** NARFL was highly expressed in HEK293T cells detected using western blot. **B, C, D**. shRNA-A and shRNA-B of five shRNA duplexes (B) induced declined *NARFL* mRNA level (C) and reduced aconitase activity (D) in HEK293T cells. **E.** Targeted indel mutations induced by CRISPR/Cas9 at the NARFL gene. The wild-type sequence was shown at the top with the target sites highlighted as blue and the mutant sequence highlighted as red. **F.** Control zebrafish (94/126) showed dominant subintestinal vessels in 3 dpfs using blood vessel staining. In NARFL knockout zebrafish (87/109), the subintestinal plexus has ectopic sprouts (*p*< 0.0001, *n*= 22/109 versus 94/126). **G.** VEGF mRNA levels were examined using qRT-PCR assay and VEGF mRNA levels were upregulated in NARFL-knockout zebrafish. **H.** The mRNA levels of SOD and CAT were declined. (*, *p*< 0.05; **, *p* < 0.01)

Among the five synthesized short hairpin RNA (shRNA) duplexes, the shRNA-A and shRNA-B knocked down NARFL expression significantly in HEK293T cells (*p* < 0.01, *n* = 3, Figure 4A, 4B, 4C). We found NARFL knockdown reduced activity of cytosolic aconitase, a Fe-S protein, significantly (both *p* < 0.01, *n* = 6, Figure 4D), but not mitochondrial aconitase and lactate dehydrogenase (LDH) activity. These indicated that NARFL knockdown might affect its function of transferring Fe-S cluster and further biogenesis of cytosolic Fe-S proteins, including aconitase in cell lines. These could be the reason for Fe^3+^ overload in the proband's lung.

### NARFL knockout led to aberrant angiogenesis in zebrafish

NARFL knockout experiments in zebrafish were performed to identify whether NARFL knockout would exhibit a vascular phenotype. As shown in Figure 4F, most of control fish at 3 days postfertilization (dpf) (n = 94/126) exhibited an orderly subintestinal vessels. However, NARFL knockout zebrafish presented ectopic subintestinal vessels sprouts (Figure 4F) (*p* < 0.001, *n* = 22/109 versus 94/126), and VEGF overexpression was also detected (Figure 4G). As shown in Figure 4G, VEGF mRNA levels of NARFL (-/-) zebrafish were significantly higher than the wild type (*p* < 0.01). In addition, the expression of superoxide dismutase (SOD) and catalase (CAT) was both declined (*p* < 0.01).

## DISCUSSION

We identified a probable causative gene *NARFL (Ser161Ile)* in a family with diffuse PAVMs, and the diffuse PAVMs were further demonstrated by positive immunostaining for platelet endothelial cell adhesion molecule-1 (CD31), CD34, cytokeratin (CK), and a-smooth muscle actin (SMA) [16]. The Ser161Ile mutation caused the mRNA instability and reduced the NARFL mutant's expression in vivo. NARFL knockdown further decreased cytosolic aconitase activity, and NARFL knockout caused aberrant subintestinal vessels in NARFL (-/-) zebrafish with VEGF overexpression. These could explain Fe^3+^ overload, upregulation of VEGF and vessels malformations in the proband's lung tissue [14-17].

Firstly, we found that Ser161Ile mutant only induced slight distance changes of hydrogen bonds in cavity of NARFL protein, and slightly influenced the hydropathicity and hydrophobicity. However, we observed there was a dosage effect of Ser161Ile mutant on *NARFL* mRNA expression, though only a decreased tendency of mRNA levels occurred among family members. This might be due to the limited sample size or other confounding factors.

Secondly, NARFL deficiency further reduced the activity of cytosolic aconitase instead of mitochondrial aconitase in NARFL-knockdown cell lines. This was understandable, since NARFL is reported to be a unique component in cytosolic iron-sulfur cluster assembly (CIA) system and functions mainly in biogenesis of cytosolic iron-sulfur protein [14]. NARFL serves as a scaffold for delivering iron-sulfur cluster to iron regulatory protein 1 (IRP1), to form cytosolic aconitase which is an important Fe-S protein [12, 14, 18]. So the deficiency of NARFL might impair the biogenesis of cytosolic Fe-S proteins and lead to inactivation of cytosolic aconitase. Furthermore, NARFL knockdown was reported to upregulate transferrin receptor 1 (involved in iron uptake) which could directly increase intracellular iron amount [12]. And cytosolic aconitase, upon loss of its iron-sulfur cluster, converts to IRP1, which could bind the 5′-untranslated regions of the ferritin heavy chain mRNA. IRP1 in turn serves to inhibit translation of ferritin heavy chain protein which is an intracellular iron storage protein, and further increases free iron levels in cells [18-19]. So the deficiency of NARFL and inactivation of cytosolic aconitase could both lead to iron uptake and storage impairment in lung tissue of the proband (as shown in Figure 3H).

Thirdly, iron overload could directly promote VEGF expression [20], which could explain the iron overload and VEGF overexpression in the proband (as shown in Figure 3J). VEGF overexpression further led to aberrant vascularization, which was confirmed by the ectopic subintestinal vessels sprouts and VEGF overexpression in NARFL(-/-) zebrafish.

In another aspect, the pathogenesis might be abnormally increased oxidative stress. NARFL was recently reported to be a key component to defend oxidative stress, companying with loss of cytosolic aconitase activity [13]. Decreased aconitase activity could in turn lead to oxidative stress through iron overload [17-18]. Iron overload activates proteins oxidation in the Fenton reaction, leading to Fe^3+^ generation and accumulation, which could be demonstrated by Prussian blue staining as shown in Figure 3H as previously reported [19]. So far, aconitase activity is widely used as a biomarker for oxidative stress, as high-intensity exercises in humans lead to inactivation of aconitase due to ROS generation [19, 21-23]. These indicated that functional aconitase was essential in maintaining redox balance and the cellular antioxidant defense system [22]. Therefore, we inferred that deficiency of NARFL promoted production of ROS, either directly [13] or via decreased aconitase activity, resulted in iron overload [19-25]. And many studies had demonstrated that increased ROS initiated pathological angiogenesis and vascular malformations via VEGF signaling pathway [26-27]. These could also be the reason for the abnormal angiogenesis in NARFL-knockout fish and the proband's lung tissue.

### Limitations

Only one family with *NARFL* mutations was found, though the cases were really rare. The complex interactions among genes were not considered in this consanguineous family. Some phenotypes except diffuse PAVMs such as interstitial pneumonitis required to be explained in the future [28].

In a word, we have identified NARFL as a possible genetic basis underlying autosomal recessive diffuse PAVMs.

## MATERIALS AND METHODS

### Patients and ethics

A consanguineous family having two daughters both diagnosed as diffuse PAVMs were recruited in Center for Gene Diagnosis, Zhongnan Hospital of Wuhan University. Investigation has been conducted according to the Declaration of Helsinki and approved by the Ethics committee of Zhongnan Hospital of Wuhan University. After informed consent was obtained from all 5 family members, 35 sporadic patients and 500 population controls, DNA and total RNA were extracted from white blood cells using the method as previous [29].

### Whole exome sequencing

WES was performed on two daughters and their parents at BGI-Shenzhen (Shenzhen, China). Exome enrichment was performed with Agilent Sure Select Human All Exon v2 kit (50Mb) (Agilent Technologies, Inc. SantaClara, CA) with an average sequencing depth of 70-fold and coverage of 97.7%. Enriched shotgun libraries were sequenced with the Illumina Hiseq2000 platform (Illumina, Inc., San Diego, CA). Raw image data and base calling were processed by Illumina Pipeline software v1.7 and SOAP aligner was used to align the high quality reads to the human reference genome (hg19). A list of qualifying genotypes was generated by filter criteria [30]. First, only protein-altering variants, such as missense variants, frame shift, indels and intron-exon boundary variants were included. Second, the variants were absent in the available four BGI-Shenzhen in-house databases. Third, the variants were also absent in the dbSNP137, HapMap, 1000 human genome dataset. Fourth, autosomal recessive models were systematically considered. Fifth, qualifying genes were checked against Uniprot (http://www.uniprot.org/) to be filtered whether it was expressed in lung or ubiquitously expressed. Sixth, ExAC (http://exac.broadinstitute.org) was used to assess the variant's frequency and influence.

### Comparative genomic hybridization

Comparative genomic hybridization (CGH) on daughters and their parents were performed in CapitalBio Cooperation (Beijing, China) to discover large deletions and copy number variations (CNVs). Human CGH 3×1.4M Whole-Genome Exon-Focused Array (Roche Diagnostics, Mannheim, Germany) was used with a median spacing of 7 kb and 5 probes/exon. Raw CGH data were extracted and analyzed using Nimble Scan v2.6 software (Roche Diagnostics) and CNVs were identified by filtering more than 5 consecutive probe segments with [log_2_ ratio]>0.25.

### Sanger sequencing of the variants

All validated variants were subsequently verified in 5 family members by Sanger sequencing. Furthermore, the mutation in NARFL gene was screened in 35 sporadic patients and 500 population controls using single strand conformation polymorphism (SSCP) analysis and confirmed using Sanger sequencing.

### In silico prediction of NARFL protein

SIFT (http://sift.jcvi.org/) and Polyphen-2 (http://genetics.bwh.harvard.edu/pph2) were used for assessing biological effects of the mutation. Multiple NARFL protein sequence alignments across species were performed using ClustalW2. SWISS-Model modeling server (http://swissmodel.expasy.org/) and ExPASy (http://www.expasy.org/tools/protparam) were used to analyze the protein structure, conserved domain and functional domain.

### Quantitative real time reverse transcript PCR

cDNA was synthesized using the Revert Aid First Strand cDNA Synthsis Kit (Fermentas, Burlington, Ontario, Canada). The expression levels of *NARFL* were detected by quantitative real time reverse transcript PCR (qRT-PCR) in a CFX 96 real time system (Bio-Rad, Shanghai, China) using SYBR mix (Applied Biosystem, Shanghai, China). The human samples were assayed in triplicate and glyceraldehydes-3-phosphate dehydrogenase (GAPDH) was used as an internal control. The mRNA expression levels of VEGF were compared between NARFL (-/-) zebrafish and controls (30 zebrafish per group) using qRT-PCR as described above. The zebrafish samples were assayed in triplicate and β-actin was used as a control. Relative gene expression levels were determined by the 2^−ΔΔCt^ method.

### Cell lines, plasmids and transfection

HEK293T, COS7, Hep3B cell lines were obtained from American Type Culture Collection (ATCC, Manassas, VA). Human umbilical vein endothelial cells (HUVECs) were obtained from Cell Bank of Shanghai Institutes for Biological Sciences. HEK293T cells were grown in Dulbecco's modified Eagle's medium and transfected with lipofectamine 2000 (Invitrogen, Shanghai, China). The pCMV-HA-wild plasmid was constructed by amplifying controls’ cDNA and the pCMV-HA-mutant plasmid was prepared using a QuikChange mutagenesis kit (Stratagene, Shanghai, China).

### The mRNA stability assay

The pCMV-HA-wild and pCMV-HA-mutant were transfected into HEK293T cells, respectively. The cells were harvested and total RNA was isolated once Actinomycin D (Sigma, USA) was added to inhibit RNA synthesis, at the indicated hours. Then NARFL mRNA level was quantified using qRT-PCR. The experiment was repeated for three times and the mean half-life of mRNA was compared by t test.

### Western blotting

Cellular protein samples were isolated using RIPAlysis buffer (Beyotime, Shanghai, China) and the protein concentration was measured by BCA assay (Beyotime). Antibodies to HA and Myc were obtained from Cell Signaling Technologies (GST, Danvers, USA). Antibody to NARFL was obtained from Abcam (Abcam, Shanghai, China). The membranes were incubated with HRP-conjugated secondary antibodies and the signals were visualized with ECL (Beyotime, Shanghai, China). The band densities of the proteins were measured with Image J software. GAPDH was selected as an internal control and the experiments were performed in triplicate.

### NARFL knockdown and enzyme assays

The pSUPER-retro-control plasmid and five shRNA duplexes were synthesized in Invitrogen (Invitrogen, Shanghai, China). Cell mitochondria isolation kit was used for the separation of mitochondria and cytosol (Beyotime), and cytochrome C was detected using western blot to guarantee no cytosolic contamination in the mitochondrial component. Aconitase activity was measured by aconitase assay kit (Sigma, Shanghai, China) in mitochondria and cytosol, as well as lactate dehydrogenase activity using LDH assay kit (Beyotime).

### Immunohistochemical staining

Lung tissues were collected from the proband and the population controls and were fixed in 10% buffered formalin. Antigen Unmasking Solution (Vector laboratories, QLD, Australia) was used for antigen retrieval and 10% horse serum was used for blocking. Sections of lung tissues were then incubated with NARFL, VEGF, CD31, CD34, SMA, and CK antibody (Abcam, Shanghai, China). The slides were then treated with secondary antibodies. Nuclei were visualized using Haematoxilin. The Fe^3+^ levels were detected using Prussian blue staining [31].

Expressions of NARFL and VEGF in lung tissues were determined using immunohistochemistry to assess the localization, intensity, and area of stained cells [32]. Intensity of the staining was graded as follows: no staining = 0, mild staining = 1, moderate staining = 2 and intense staining = 3. The area of staining was scored on the following scales: no stained cells in any microscopic field = 0, < 25% of lung cells stained positively = 1, 25%-50% = 2, 50%-75% = 3 and > 75% = 4. The sum of the scores for area and intensity of staining were used for statistical analysis. At least six fields were randomly collected from each slide and were analyzed by Image J software.

### Zebrafish model

All experiments involving zebrafish were performed in accordance with Chinese Legislation for Animal Experimentation and European Communities Council Directive 86/609/EEC. When required for observing phenotypes of live embryosno later than 5-dpf, embryos were anaesthetized by the addition of tricaine methanesulfonate to the embryo medium to a concentration of 150 mg/L. Narfl(zebrafish)-knockout experiments were performed using clustered regularly interspaced short palindromic repeat (CRISPR)/Cas9 genome editing technology in zebrafish as previous [33]. A guide RNA (gRNA) was generated as described [34]. gRNA scaffold was cloned into pMD 19-T vector (TakaRa; cat# D102A). Double-stranded DNA for specific gRNA synthesis was PCR amplified (Transgen; cat# AS211) using the primers: Forward: 5’- TAATACGACTCACTATAGGGACATGCAGACGCCAACATGTTTTAGAGCTAGAAATAGC-3’, Reverse: 5’-AGCACCGACTCGGTGCCACT-3’. After gel extraction, gRNA was synthesized using T7 RNA Polymerase (NEB; cat# M0251S). Subsequently, 100pg gRNA and 150pg Cas9 mRNA (SBI, CAS500A-1) were injected into one or two-cell stage zebrafish embryos. Following hatching, 10~20 embryos were collected for DNA extraction. The target gene region was amplified using the primers: Forward: 5’- AGACTCAAGTCAGAAGCCCTA-3’, Reverse: 5’- CG TTCTTATTTTGACAGCAGCAC-3’. Gene sequencing was used to detect and characterize the mutation of narfl. The remainder of the embryos were raised up to adults as F0 and mated with wild-type zebrafish to generate the F1 generation. The F1 adults were genotyped by DNA sequencing of the PCR products amplified from their caudal fin. F1 strains harboring the detected mutations were crossed to each other to obtain the F2 generation. Blood vessels of zebrafish were stained using alkaline phosphatase staining [33].

### Statistics

Results were expressed as mean ± standard deviation. One-way ANOVA (F-test) was carried out for comparing vascular phenotypes of zebrafish between wild and mutant types. Comparison between two groups was carried out using the Student's test (t-test) such as mRNA and protein levels. Correlations in mRNA stability were assessed using the Karl Pearson coefficient of correlation. For all tests, a *p* - value of ≤ 0.05 with two-tailed was considered to be of statistical significance.

## SUPPLEMENTARY MATERIALS TABLES






