# Supplementary material for: A novel mutation in nuclear prelamin a recognition factor-like causes diffuse pulmonary arteriovenous malformations
Source: Oncotarget. 2016 Nov 7;8(2):2708–18. doi: 10.18632/oncotarget.13156 (PMC5356835; doi:10.18632/oncotarget.13156)
Supplement: Supplementary file 1 [file oncotarget-08-2708-s001.pdf]

A novel mutation in nuclear prelamin a recognition factor-like causes diffuse pulmonary arteriovenous malformations

SUPPLEMENTARY TABLES

Supplementary Table S1: The entire list of candidate variants in WES.

See Supplementary File 1

Supplementary Table S2: The entire list of copy number variants in CNVs experiments.

See Supplementary File 2
